# Yves Moreau has received the 2023 Einstein Foundation Individual Award for Promoting Quality in Research

**DOI:** 10.1093/bioadv/vbae039

**Published:** 2024-03-29

**Authors:** Thomas Lengauer

**Affiliations:** Max Planck Institute for Informatics, Saarland Informatics Campus, 66123 Saarbrücken, Germany

On 14 March 2024, Yves Moreau has received the 2023 Einstein Foundation Individual Award for Promoting Quality in Research.

Yves Moreau is a professor of engineering at the University of Leuven in Belgium. Trained as an engineer, he turned to computational biology at the beginning of the new millennium. Together with his team, he is developing data fusion methods for the identification of candidate disease genes and variants (Nitsch *et al.* 2010, Sifrim *et al.* 2013, Tranchevent *et al.* 2016), especially in rare genetic diseases. He also investigates data fusion methods for drug design and drug discovery (Laenen *et al.* 2013, Parisi *et al.* 2020). A third focus topic of his work is federated and privacy-preserving analysis of chemical or clinical genetic data (Simm *et al.* 2021, Heyndrickx *et al.* 2023a,b). Towards these purposes, he employs methods of machine learning, such as Bayesian matrix factorization and deep learning. Going beyond mere methodical work, he strives for clinical or industrial applicability of his methods. Thus, he cofounded the company Cartagenia, which focused on bioinformatical solutions for clinical genetic diagnosis and has since become part of Agilent Technologies.

His research transcends technical aspects and reaches into the ethical use of the relevant methods and data. It is the attention that Yves Moreau pays to the ethical aspect of doing science which has been acknowledged by the award. The Einstein Foundation Award for Promoting Quality in Research has been conferred annually since 2021. The award honors individual researchers as well as organizations from all fields of science that have made substantial contributions to fostering the increase of the quality and reliability of research. Each year a senior and a junior researcher as well as an organization or group are honored. Yves Moreau has received the Senior Individual Award for 2023, which is worth €200,000.

The jury statement lauding the award reads: ‘Yves Moreau forcefully advocates for ethical standards in the utilization of human DNA data in research and privacy-preserving analysis of clinical genetic data in the age of artificial intelligence and big data’.

The basis for the conferral of the award is twofold. On the one hand, Yves’ work of developing privacy-preserving methods for analyzing genetic data was commended. A decade ago, he pioneered the concept of federated analysis of genomic data, where sensitive data is analyzed through the aggregation of local statistical analysis rather than centralizing it. More recently, he contributed to further strengthening these methods by combining them with cryptographic techniques. Federated data analysis is now being increasingly deployed to support compliance with privacy legislation, such as the EU General Data Protection Regulation. On the other hand, Yves has been active for a long time in raising awareness regarding the ethical use of genetic data. As a member of the Board of Directors of International Society for Computational Biology (ISCB), of which he is also a fellow, he has striven for appropriate codes for accepting sponsorship from industry (e.g. excluding companies whose business model demonstrably harms public health). Furthermore, he has drawn attention to cases in which genetic data, in particular for forensics, have been used for research without appropriate ethical safeguards, such as consent of the involved individuals or concern for the risks of misuse against vulnerable communities (Moreau 2019, Forzano *et al.* 2021, Lewis 2024).

We have asked Yves a few questions on the occasion of the conferral of the award.

**Q: Your field of research is computational biology. In the past you have been especially troubled with problematic handling of genomic sequence data, in the sense that the concerns of the data donors in terms of privacy and informed consent are not adequately addressed. Which challenges do you see along the road to rectifying this situation?**

The specific area on which I focus is forensic population genetics, which combines the techniques of forensic genetics for human DNA identification with studies of genetic diversity across different populations. Forensic genetic kits allow the amplification and sequencing several loci across the genome and are used to match crime scene DNA samples to suspects or people registered in DNA databases. Because DNA matching depends on statistics of allele frequencies in each population, the same DNA match leads to different statistical evidence in different populations. Forensic genetic kits thus need to be statistically calibrated for the populations in which they will be used. However, DNA databases created using such tools are not always used simply for legitimate law enforcement purposes but sometimes recklessly deployed as part of an arsenal of mass surveillance and social control, often along other technologies such as camera surveillance, facial recognition, and internet censorship. In such circumstances, research that clearly enables the development and deployment of this technology in those settings becomes ethically questionable: Were research participants adequately informed of the risks associated with the research? Were they free to decline participation without fear of retaliation? Did researchers engage with their communities to assess the potential risks and benefits of the research for these communities? Have the potential harms linked to such research been adequately minimized?

On this basis, I have raised concerns with over 100 research articles, mostly in forensic population genetics. In many cases, answers to these questions were found to be lacking. This has led to the retraction of 28 research articles on forensic genetics (and one on facial recognition), so far. However, the process for the ethical reassessment of these articles has been painfully slow, with multiple cases still under assessment by publishers after more than three years. While some publishers, editors-in-chief, and editorial boards have handled cases diligently, in many other cases publishers and editors-in-chief have been uncooperative or even obstructive. It unfortunately suggests that the research integrity process of academic publishers is subordinate to their short-term interests. It often seemed that high-profile media exposure in major news media, such as The New York Times or The Guardian, or in major scientific journals, such as Nature and Science, or pressure by powerful research or governmental institutions was needed to obtain these retractions, rather than the research integrity process naturally following its course. The question is whether a tipping point has now been reached where publishers will be forced to finalize their decisions regarding the remaining 70+ cases. The entire pool of ethically problematic articles in forensic genetics is unfortunately much larger with several hundred other articles that could still be challenged.

**Q: Your work on federated computing is aimed at facilitating data analysis without divulging protected information, such as on (i) genomes or (ii) chemical compounds when analyzing the respective data. The beneficiaries from your technology are patients who are concerned about privacy, in the first case, and chemical industry which is concerned about intellectual property, in the second case. Do you see the same or different challenges when addressing the concerns of these two quite different target groups?**

While a variety of techniques can be brought to bear on these problems, they do not differ so much between the application areas. I view such techniques—ranging from simple statistical aggregation to (partially) homomorphic encryption and secure multiparty computation going over differential privacy—as a toolbox from which you select techniques depending on the specific context of the application while considering tradeoffs between enhanced security and increased computation cost. One important lesson learned is that there do not yet appear to be techniques that can offer full protection and be entirely secure from attacks while also being able to deliver all essential analysis results. It seems that, for the moment, such federated analysis will be more like IT security where attacks are always a possibility but where systems can be sufficiently hardened against a given threat, at a cost commensurate to the threat. Another important lesson is that even when technical solutions can be offered that provide adequate protection, social issues in consortium building and trust between partners remain paramount. It is not just a question of whether data privacy and confidentiality are adequately protected, but also of who can do what with which data and how partners feel other partners could exploit their data. Nevertheless, federated analysis models will be a key strategy to deal with the fragmented international landscape of privacy regulation.

**Q: What are concerns you have regarding ethical approaches to science transcending your primary research field? Which of those concerns do you plan to address in the future?**

What I have seen in my work is that our approaches to research ethics fall short. While many of us endure the pains and delays of bureaucratic processes to get their research approved or obtain access to data, I have seen how these processes can fail where they matter the most. This is incredibly frustrating. While ethics and regulation have become important issues across all scientific fields, I feel that, in practice, research ethics is too often seen as an impediment to scientific productivity and that it is easily overcome by conflicts of interests at all levels. Ethics and regulation cannot work as intended if they do not feed into a strong culture of ethics and responsibility.

During the past half century, science has been increasingly focused on academic productivity and economic value. A bit as a caricature, we could say that scientists have been expected to be objective, stay in their labs, and not interfere with world affairs. This is not the only model. A case in point is how, after World War II, some of the world’s best scientists, from Bertrand Russell to Albert Einstein, engaged with society to limit nuclear proliferation. Science shapes society and its future. Therefore, scientists have a duty to engage with society and contribute to the debates about how technology gets deployed in society. The recent advances in artificial intelligence have made this need even more pressing. However, many scientists, in particular engineers and computer scientists, lack the motivation and culture needed to step into such debates, while our expertise is crucial if informed and reasonable societal choices are to be made. Many of us—including myself for several decades—simply prefer to solve challenging problems rather than engage with the messiness of social debates. Most of us do not realize how our minute contributions mesh with those of other scientists to profoundly impact society. We do not know how to talk about technology and societal issues. We often know too little about ethics, law, philosophy, epistemology, sociology, and history to reflect critically about our work and to feel empowered to choose for ourselves which technology we want to develop and to what end.

In the future, I want to tackle these issues by collaborating with experts both in STEM fields and in the social sciences to promote awareness of these issues and develop tools to educate students, researchers, and people with a scientific background on these issues. Influencing the culture and attitudes of people developing technology provides enormous leverage. I am incredibly encouraged by the interactions I have with my students, who are from a generation that is unfortunately faced with the huge challenges of climate change and environmental sustainability and realizes that business as usual is not an option. They absolutely crave such intellectual tools that will empower them to shape a fairer and more sustainable future.

**Yves, thanks much for your insightful comments and congratulations to your impressive award.**

*The interview was conducted by Thomas Lengauer.*

## Data Availability

No new data were generated or analysed in support of this research.

## References

[vbae039-B1] Forzano F , Genuardi M, Moreau Y et al; European Society of Human Genetics. ESHG warns against misuses of genetic tests and biobanks for discrimination purposes. Eur J Hum Genet 2021;29:894–6. 33456053 10.1038/s41431-020-00786-6 PMC8187659

[vbae039-B2] Heyndrickx W , Arany A, Simm J et al Conformal efficiency as a metric for comparative model assessment befitting federated learning. Artifi Intelli Life Sci2023a;3:100070.

[vbae039-B3] Heyndrickx W , Mervin L, Morawietz T et al MELLODDY: cross-pharma federated learning at unprecedented scale unlocks benefits in QSAR without compromising proprietary information. J Chem Inf Model2023b. 10.1021/acs.jcim.3c00799 PMC11005050 37642660

[vbae039-B4] Laenen G , Thorrez L, Börnigen D et al Finding the targets of a drug by integration of gene expression data with a protein interaction network. Mol Biosyst 2013;9:1676–85. 23443074 10.1039/c3mb25438k

[vbae039-B5] Lewis D. Unethical studies on Chinese minority groups are being retracted—but not fast enough, critics say. Nature 2024;625:650–4. 38267675 10.1038/d41586-024-00170-0

[vbae039-B6] Moreau Y. Crack down on genomic surveillance. Nature 2019;576:36–8. 31796907 10.1038/d41586-019-03687-x

[vbae039-B7] Nitsch D , Gonçalves JP, Ojeda F et al Candidate gene prioritization by network analysis of differential expression using machine learning approaches. BMC Bioinformatics 2010;11:460. 20840752 10.1186/1471-2105-11-460 PMC2945940

[vbae039-B8] Parisi D , Adasme MF, Sveshnikova A et al Drug repositioning or target repositioning: a structural perspective of drug-target-indication relationship for available repurposed drugs. Comput Struct Biotechnol J 2020;18:1043–55. 32419905 10.1016/j.csbj.2020.04.004 PMC7215100

[vbae039-B9] Sifrim A , Popovic D, Tranchevent L-C et al eXtasy: variant prioritization by genomic data fusion. Nat Methods 2013;10:1083–4. 24076761 10.1038/nmeth.2656

[vbae039-B10] Simm J , Humbeck L, Zalewski A et al Splitting chemical structure data sets for federated privacy-preserving machine learning. J Cheminform 2021;13:96. 34876230 10.1186/s13321-021-00576-2 PMC8650276

[vbae039-B11] Tranchevent L-C et al Candidate gene prioritization with endeavour. Nucleic Acids Res 2016;44:W117–21. 27131783 10.1093/nar/gkw365 PMC4987917

